# Intraoperative kinematics of the bicruciate retaining TKA using functional alignment and their influence on the clinical outcomes

**DOI:** 10.1186/s42836-025-00319-w

**Published:** 2025-07-03

**Authors:** Hiroshi Inui, Ryota Yamagami, Kenichi Kono, Kohei Kawaguchi, Haruhiko Nakamura, Kazuo Saita, Shuji Taketomi, Sakae Tanaka

**Affiliations:** 1 Saitama Medical Center, Saitama Medical University, 1981 Kamoda, Saitama, Kawagoe City 350-8500 Japan; 2 Department of Orthopaedic Surgery, Faculty of Medicine, The University of Tokyo, Tokyo, 113-8654 Japan

**Keywords:** Total knee arthroplasty, Functional alignment, Knee kinematics, Clinical outcomes

## Abstract

**Background:**

Our surgical team has been performing bicruciate-retaining total knee arthroplasty (BCR-TKAs) with functional alignment (FA). This study aimed to investigate knee kinematics before and after FA BCR-TKA, as well as the influence of these changes on clinical outcomes.

**Methods:**

Fifty cases of BCR-TKAs were included. Intraoperative rotational kinematics and anteroposterior translations between before (preinsertion group) and after (postinsertion group) BCR-TKA were compared. The relationship between clinical outcomes and intraoperative kinematic parameters between the two groups was evaluated.

**Results:**

The tibial internal rotational angles of the preinsertion group were significantly larger than those of the postinsertion group at 0°, 60°, 90°, and maximum flexion angles. Anteroposterior (AP) translation of the femur center relative to the tibial center of the preinsertion group was significantly smaller than that of the postinsertion group at 60° and 90° of flexion angles. No difference was found between the two groups at 0°, 30°, and maximum flexion angle. A negative relationship was found between the difference in rotational angles at maximum flexion and knee injury, and osteoarthritis outcome score (KOOS) activity of daily living (ADL), and improvement of KOOS symptom and ADL subscale scores. A positive relationship was found between the difference in rotational angles at 0° and improvement of KOOS pain, sports, and quality of life subscale scores.

**Conclusions:**

AP translation of the femur after BCR-TKA with respect to the tibia was similar to that of the preoperative knee. The change in rotational knee kinematics after BCR-TKA showed associations with clinical outcomes; however, the relationship remains multifactorial and should be interpreted with caution.

## Introduction

Total knee arthroplasty (TKA) is the gold standard of treatment for late-stage osteoarthritis (OA). The rates of dissatisfaction after TKA are often reported as around 20%, although newer studies suggest considerably less dissatisfaction [1–4].

Bicruciate-retaining (BCR) prostheses, which preserve both the anterior and posterior cruciate ligaments (ACL and PCL, respectively), have been introduced to recreate normal knee movements. However, BCR-TKA is still being debated among orthopedic surgeons because most studies on BCR-TKAs have shown clinical outcomes similar to other ACL-sacrificing TKAs, and several studies have reported a high complication rate, including stiffness and early revision [5–9]. Our surgical team has been performing BCR-TKA with functional alignment (FA), which is a modification of kinematic alignment. FA BCR TKA aims to achieve balanced flexion and extension gaps using computer tools, including the navigation and the robot system [10–12]. Recently, we showed the excellent clinical outcomes of FA BCR-TKA [13].

However, regarding the kinematics of BCR-TKA, no consensus has been established as to whether BCR-TKA reproduces normal knee kinematics [14–16]. Theoretically, FA BCR-TKA reproduces normal-like knee kinematics by preserving all knee ligaments and reproducing constitutional alignment. However, no studies have evaluated the kinematics after FA BCR-TKA.

We hypothesized that the FA BCR TKA would reproduce the preoperative knee kinematics and that the smaller changes in kinematics would lead to better clinical outcomes. Therefore, this study aimed to investigate the knee kinematics and the influence of the change in knee kinematics between before and after FA BCR-TKA on the clinical outcomes.

## Methods

This study was approved by the review board of the institution (No.10462). All patients provided written informed consent.

**Fig. 1:**
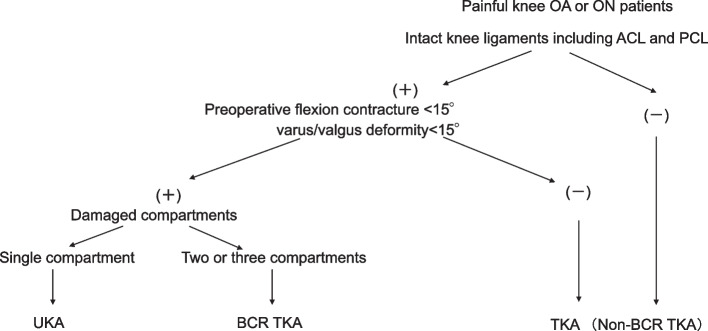
Indications of BCR TKA

Between January 2019 and March 2022, out of a total of 520 knee arthroplasty procedures (405 TKAs, 115 UKAs), 69 BCR TKAs (Journey II XR; Smith and Nephew, Memphis, TN, USA) were performed using an image-free navigation system (Precision N; Stryker Orthopedics, Mahwah, NJ, USA). The surgical indications for BCR TKAs were knee OA or osteonecrosis (ON) of more than two compartments, intact cruciate and collateral ligaments, preoperative flexion contracture < 15°, and preoperative deformity < 15° (Fig. 1). In this study, 50 BCR-TKAs met the following inclusion criteria: (1) varus deformity (2) complete data entry, and (3) minimum follow-up period of 1 year. Nine BCR-TKAs were performed for preoperative valgus deformity patients; 5 BCR-TKAs’ preoperative data were incomplete, and 4 BCR-TKAs were lost to follow-up. The competency of the ligaments was ascertained by manual stress and magnetic resonance imaging.

Fifty BCR-TKAs were performed in 48 patients. Twelve were male and 36 were female. The average age was 72.4 ± 7.4 years, and the body mass index was 25.6 ± 3.4 kg/m^2^. The diagnosis was OA in 42 knees and ON in eight knees. The preoperative average hip-knee-ankle angle was 173.3° ± 2.9°.

Preoperative patient demographics, including range of motion (ROM), preoperative clinical scores obtained using the validated version of the knee injury and osteoarthritis outcome score (KOOS) [17, 18]. The KOOS is a self-reported questionnaire with 42 items in five separately analyzed subscales of pain, symptoms, activities of daily living (ADL), physical, sports, and recreational function, and knee-related quality of life (QOL). Each of the five scores was calculated as the sum of the items included, and the scores were then transformed into a 0- to 100-point scale, with 0 points representing extreme knee problems and 100 points representing no knee problems. And the KOOS is a valid, reliable, and responsive outcome measure for TKA patients [19, 20]. The satisfaction score of the 2011 Knee Society Scoring (KSS) system [21] was recorded (Table 1).

**Table 1:** Clinical data before and after BCR TKA

	Before BCR-TKA	After BCR-TKA	*P*-value
Maximum extension (°)	− 3.1 ± 3.4	− 0.6 ± 1.4	< 0.001
Maximum flexion (°)	130.8 ± 8.1	128.1 ± 8.1	0.07
KOOS			
Pain	48.5 ± 18.8	89.6 ± 10.1	< 0.001
Symptom	60.5 ± 17.7	87.2 ± 9.8	< 0.001
ADL	58.3 ± 18.8	88.6 ± 9.6	< 0.001
Sports	24.4 ± 23.0	58.6 ± 24.4	< 0.001
QOL	28.5 ± 18.0	77.0 ± 17.6	< 0.001
2011KSS			
Satisfaction	12.5 ± 5.8	31.3 ± 7.4	< 0.001

ADL, activities of daily living; BCR, bicruciate retaining; KOOS, knee injury and osteoarthritis outcome score; KSS, Knee Society Score; QOL, quality of life; TKA, total knee arthroplasty; UKA, unicompartmental knee arthroplasty

Four knee surgeons performed all procedures using the same surgical technique. A senior surgeon (H.I.) participated in all procedures either as the chief surgeon or the first assistant.

### Surgical procedure

**Fig. 2:**
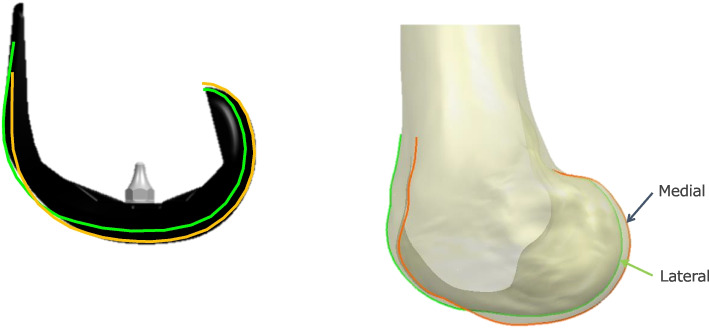
Journey II TKA system. Sagittal view of the femoral component

All the surgical procedures and intraoperative measurements were performed using the tourniquet. In all patients, a paramedian approach was used, and the patella was not everted. The surgeon performed aggressive removal of osteophytes and minimal release of medial soft tissues for bone resection. On the femoral side, measured resection was performed using navigation after estimating cartilage wear. The femoral component design of the Journey TKA system was asymmetric (Fig. 2), and the medial and lateral distal condyles were 9.5 and 7 mm thick, respectively. Therefore, using the navigation system, distal (thickness, 7–8 mm) resection was performed on the medial side, considering the cartilage wear (1–2 mm) based on the intraoperative findings. In cases of complete cartilage loss, 2 mm was assumed as the missing cartilage thickness following previous studies [22], while for partial loss, 1 mm was assumed as the missing cartilage thickness, and osteotomy was performed [10]. Femoral alignment in the sagittal plane aimed at 4° of flexion to avoid femoral cortex notching [23].

Proximal tibial resection was then performed. A distal femoral spacer block mimicking the distal end of the femur component (medial 9.5 mm, lateral 7 mm) was placed on the resected distal femur, and the knee was brought into extension. Varus–valgus stress was applied to evaluate the medial and lateral joint laxity using the navigation system, and the amount of tibia cut was decided considering these laxities [12]. The tibial design of the Journey TKA system was also asymmetric. The thickness of the thinnest tibial insert was 8.5 mm for the medial side and 11 mm for the lateral side. Therefore, for the varus knee, the amount of the bone resection of the lateral tibial plateau was set at 11 mm, and the amount of the resection of the medial side varied from 5 to 9 mm according to the soft tissue balance at this time point. In the sagittal plane, with the use of the navigation system, we reproduced a native slope in patients with a posterior tibial slope of < 10°. In patients with a posterior tibia slope of > 10°, we reduced the posterior slope so as not to exceed 10° [24, 25]. Rotational orientation of the tibial component was decided considering Shakespeare’s line [26] and the substitute anteroposterior (AP) line [27]. The extension and flexion gaps were measured using a force-controlled, compartment-specific ligament tensioner with a distraction force of 80 N for each of the medial and lateral compartments. For the posterior femur resection, the amount of resection was adjusted to make the extension and flexion gaps of the medial and lateral compartments equal, allowing for a slight lateral ligamentous laxity [28]. For instance, if the joint gap at extension and flexion was 21 mm and 13 mm, respectively, in the medial compartment and 23 mm and 16 mm, respectively in the lateral compartment before the femoral posterior resection, we adjust the cutting amount and the rotation of posterior reference cutting guide to cut 8 mm off the posterior medial femoral condyle and 7 mm off the posterior lateral femoral condyle.

### Intraoperative evaluation

The kinematics of the knee before implantation was evaluated after exposing the knee joint, removing osteophytes, and minimal release of medial soft tissues for bone resection. The tracker pins were placed, and the navigation system was registered according to the manufacturer’s instructions. After registration, the kinematics of the preoperative knee were measured using the navigation system from extension to flexion. The dissected capsule was temporarily closed with a suture. To record the position at maximum extension, the extension movement initially began with the heel supported. While supporting the heel with an open palm and touching the thigh with the opposite hand, the surgeon gently flexed the knee to its final points, and the knee flexion was assisted by gravity [29]. The kinematics were measured three times, twice by the chief surgeon and once by the first assistant, who did not see the navigation monitor when the chief surgeon evaluated the kinematics. Inter-class reliabilities of the rotational alignment at each flexion angle were 0.84–0.92, and inter-class reliabilities of the AP position were 0.85–0.94. And intra-class reliabilities of the rotational alignment at each flexion angle were 0.80–0.88, and intra-class reliabilities of the AP position were 0.82–0.88. The intra-class and inter-class reliability values were all > 0.8, indicating strong reliability. To keep the positional relationship between the femur and the tibia, the rotation angle at 0° between the femur and the tibia of the preoperative knee was defined as the zero position for each case. After the final implantation and the dissected capsule was closed with a suture, and the kinematics of the knee after BCR TKA were also evaluated.

Using the navigation kinematic data obtained during the motion cycles, rotation patterns from maximum extension to maximum flexion (flexion angles at 0°, 30°, 60°, 90°, and maximum flexion) were evaluated in each patient. A positive value indicates internal rotation of the tibia relative to the femur. The AP translation of the femur on the tibia in the sagittal plane from full extension to maximum flexion was also evaluated by comparing the relative locations of the femoral and tibial centers registered in the navigation system. The position of the femoral center relative to the tibial center of the preoperative knee at 0° was defined as zero for each case. A positive value indicates the anterior position of the femoral center relative to the tibial center.

### Postoperative rehabilitation

The same rehabilitation protocols were applied to all patients. ROM exercise and walking exercise with a crutch and then a walker were started on the first postoperative day. At 2–3 weeks postoperatively, the patient was discharged from our hospital and completed their rehabilitation protocol with physiotherapists.

### Postoperative evaluation

The clinical outcomes of BCR TKAs were evaluated in terms of the ROM, KOOS, and the satisfaction score of the 2011 KSS at one year postoperatively.

### Statistical analysis

Data were analyzed using the Bell Curve 2016 (SSRI Co., Ltd., Tokyo, Japan) software package for Microsoft Windows, and normality and distribution were assessed using the Kolmogorov–Smirnov test. The clinical parameters and intraoperative kinematic parameters were compared between the two groups (preinsertion group, after BCR-TKA; postinsertion group, before BCR-TKA) using the paired *t*-test. Group A was defined as the preinsertion group, and Group B as the postinsertion group. The correlation between the clinical outcomes and the difference in kinematic parameters, and the correlation between the degree of improvement in the clinical parameters and the difference in kinematic parameters between the two groups were evaluated. According to the statistical power analysis using G*Power 3 (Heinrich Heine Universität Düsseldorf, FRG), the estimated sample size was 46 [30]. All significance tests were two-tailed, and a significance level of *P* < 0.05 was used for all tests.

## Results

### Clinical outcomes

The clinical outcomes 1 year postoperatively are shown in Table 1. The extension angle improved significantly after BCR-TKA from − 3.1° ± 1.9° to −0.6° ± 1.9° (*P* < 0.001). Satisfaction score of KSS 2011 improved significantly from 12.5° ± 5.8° to 31.3° ± 7.4° (*P* < 0.001). KOOS scores improved significantly in all subscales. KOOS Pain score improved from 48.5 ± 18.8 to 89.6 ± 10.1 (*P* < 0.001), KOOS Symptom score improved from 60.5 ± 17.7 to 87.2 ± 9.8 (*P* < 0.001), KOOS ADL score improved from 58.3 ± 18.8 to 88.6 ± 9.6 (*P* < 0.001), KOOS Sports score improved from 28.5 ± 18.0 to 77.0 ± 17.6 (*P* < 0.001).

### Rotational kinematics

**Fig. 3:**
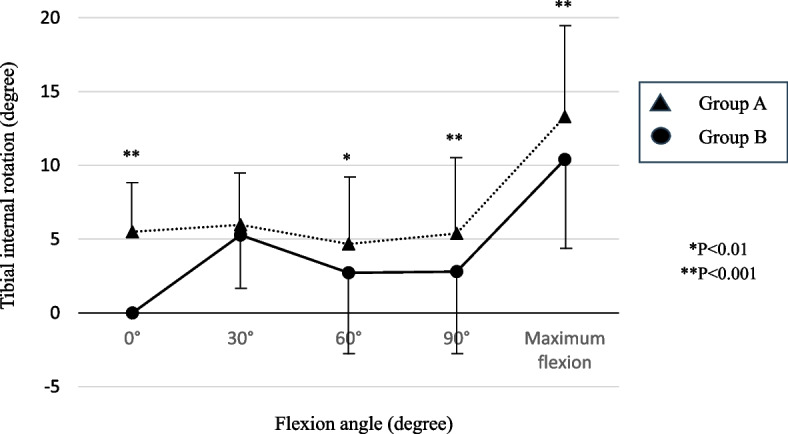
Tibial internal rotational angle relative to the femur at each flexion angle. A significant difference was observed at 0°, 60°, 90°, and maximum flexion angles

Both groups showed a gradual increase in tibial internal rotation during flexion between 0° and 30° and > 90° and a gradual decrease between 30° and 60° of flexion. The tibial internal rotational angles of group A were significantly larger than those of group B at 0°, 60°, 90°, and the maximum of flexion angles (at 0°, 90° and maximum angle: *P* < 0.001; at 60°: *P* = 0.002) (Fig. 3).

### AP translation of the femur to the tibia

**Fig. 4:**
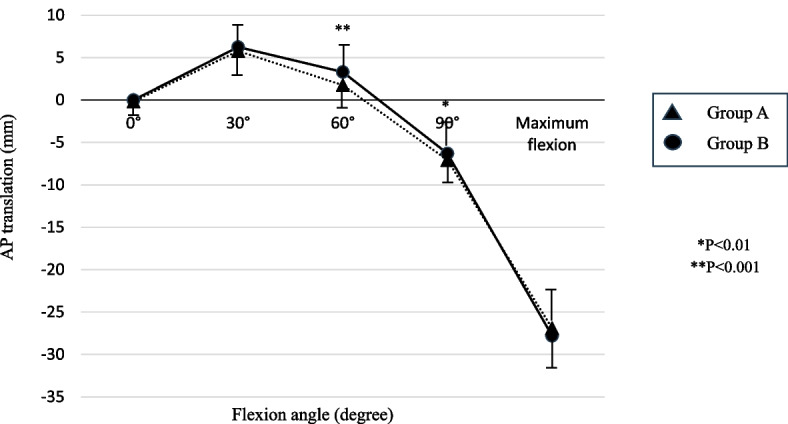
Comparison of the anteroposterior translation of the femur relative to the tibia. A significant difference was seen at 60° and 90° of flexion

Both groups showed anterior translation between full extension and 30° of flexion and posterior translation during flexion > 30°. The femoral center of group A was significantly more posteriorly placed than that of group B at 60° and 90° of flexion angles (60°, *P* < 0.001; 90°, *P* = 0.005). No statistically significant difference was found between the two groups at 0°, 30°, and maximum flexion angle (Fig. 4).

### Difference in the rotational angles between groups A and B

**Fig. 5:**
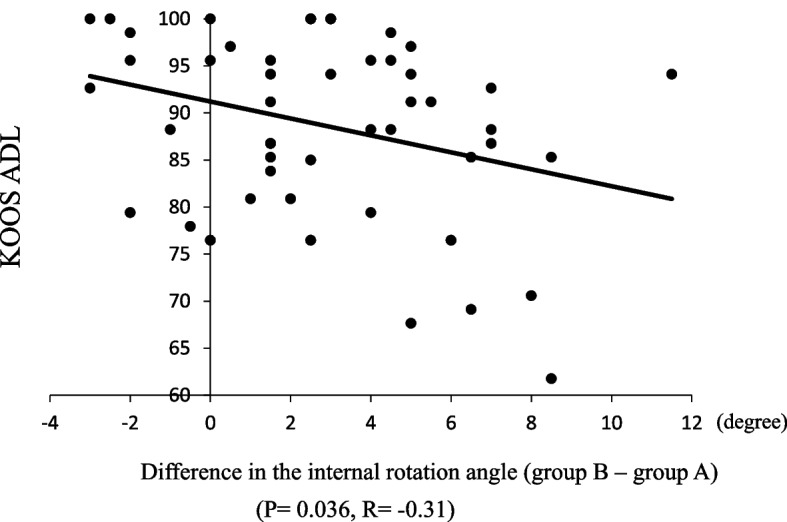
Correlation between the difference in rotational angles at maximum flexion and the KOOS ADL subscale (R =  − 0.31, *P* = 0.036)

**Fig. 6:**
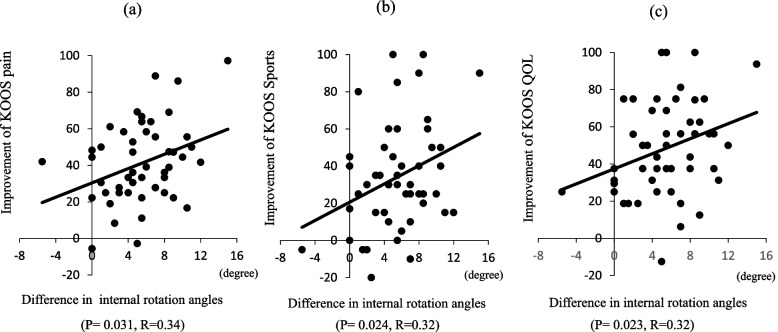
Correlation between the difference in rotational angles at 0° and improvements in the KOOS subscales of pain, sports, and quality of life (QOL). **a** The difference in rotational angles positively correlated with the degree of improvement in the KOOS ADL subscale score (R = 0.34, *P* = 0.031). **b** The difference in rotational angles positively correlated with the degree of improvement in the KOOS sports subscale score (R = 0.32, *P* = 0.024). (c) The difference in rotational angles positively correlated with the degree of improvement in the KOOS QOL subscale score (R = 0.32, *P* = 0.023)

**Fig. 7:**
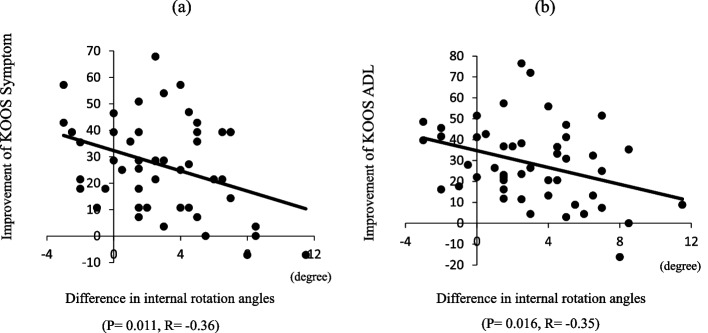
Correlation between the difference in rotational angles at maximum flexion and improvements of KOOS symptom and ADL subscale scores. **a** The difference in the rotational angles negatively correlated with the degree of improvement in the KOOS symptom subscale score (R =  − 0.36, *P* = 0.011). **b** The difference in rotational angles negatively correlated with the degree of improvement in the KOOS ADL subscale score (R =  − 0.35, *P* = 0.016)

The difference in the rotational angles between the two groups (Group B and Group A) at 0°, 30°, 60°, 90°, and maximum flexion were 5.6° ± 3.9°, 0.7° ± 3.7°, 1.9° ± 4.3°, 2.6° ± 4.2°, and 2.9° ± 3.3°, respectively. The correlation coefficients between the variations in the difference and postoperative clinical outcomes are shown in Table 2. A negative relationship was found between the difference in rotational angles at maximum flexion and the score of the KOOS ADL subscale (*P* = 0.036) (Fig. 5). The correlation coefficients between the difference in rotational angles and the improvement of postoperative clinical outcomes are shown in Table 3. A positive relationship was observed between the difference in rotational angles at 0° and improvement in the scores of the KOOS subscales of pain (*P* = 0.031), sports (*P* = 0.024), and QOL (*P* = 0.023) (Fig. 6). A negative relationship was also found between the difference in rotational angles at maximum flexion and improvement of scores of the KOOS subscales of symptom (*P* = 0.011) and ADL (*P* = 0.016) (Fig. 7).

**Table 2:** Correlation coefficients between variations in rotational angles and postoperative clinical outcomes of bicruciate-retaining total knee arthroplasty

	0°	30°	60°	90°	Max
Pain	< 0.01	−0.2	−0.13	−0.09	−0.24
Symptom	0.04	−0.09	0.01	0.03	−0.27
ADL	0.15	−0.07	0.05	0.09	−0.31*
Sports	0.24	0.11	0.15	0.12	−0.06
QOL	0.19	0.08	0.11	0.1	−0.04
Satisfaction	0.05	0.1	0.07	0.14	−0.13

ADL, activities of daily living; QOL, quality of life

^*^Correlations are statistically significant (*P* < 0.05)

**Table 3:** Correlation coefficients between variations in rotational angles and improvements in the clinical outcomes of bicruciate-retaining total knee arthroplasty

	0°	30°	60°	90°	Max
Pain	0.34*	0.05	−0.09	0.15	−0.13
Symptom	0.26	0.16	0.05	0.13	−0.36*
ADL	0.23	0.13	0.02	0.03	−0.35*
Sports	0.32*	0.16	0.07	0.06	−0.16
QOL	0.32*	0.08	0.12	0.07	−0.17
Satisfaction	0.3	0.09	−0.03	−0.14	−0.01

ADL, activities of daily living; QOL, quality of life

^*^Correlations are statistically significant (*P* < 0.05)

### Difference in the AP translation between groups A and B

The difference in the AP translation between the two groups (Group B and Group A) at 0°, 30°, 60°, 90°, and maximum flexion was 0.2 ± 2.2, 0.4 ± 2.1, 1.5 ± 2.2, 0.9 ± 1.8, and − 0.3 ± 3.2 mm, respectively. The correlation coefficients between the difference in AP translation at each flexion angle and postoperative clinical outcomes are shown in Table 4. No relationship was observed between the difference in AP translation and clinical outcomes at 1 year postoperatively. The correlation coefficients between the difference in the AP translation at each flexion angle and the degree of improvement in the clinical outcomes are shown in Table 5. No relationship was found between the difference in AP translation and clinical outcomes at 1 year postoperatively.

**Table 4:** Correlation coefficients between variation of anteroposterior translation and clinical outcomes of bicruciate-retaining total knee arthroplasty

	0°	30°	60°	90°	Max
Pain	−0.15	−0.13	−0.14	−0.06	−0.09
Symptom	0.03	< 0.01	−0.02	−0.14	−0.02
ADL	0.03	< 0.01	0.04	−0.01	0.07
Sports	−0.04	−0.08	0.14	−0.05	−0.07
QOL	0.03	0.07	0.07	<0.01	−0.09
Satisfaction	−0.06	0.02	0.13	−0.03	−0.08

ADL, activities of daily living; QOL, quality of life

**Table 5:** Correlation coefficients between variations in anteroposterior translation and improvements in the clinical outcomes of bicruciate-retaining total knee arthroplasty

	0°	30°	60°	90°	Max
Pain	0.01	0.11	−0.04	−0.03	−0.01
Symptom	−0.07	0.09	0.14	0.29	0.01
ADL	−0.06	0.04	0.09	0.2	0.11
Sports	0.04	0.08	0.13	0.2	0.11
QOL	0.08	0.01	0.03	0.03	−0.06
Satisfaction	−0.07	0.01	0.02	−0.02	−0.09

ADL, activities of daily living; QOL, quality of life

## Discussion

In this study, one of the most important findings is that the AP translation of the femur relative to the tibia after BCR-TKA was comparable to that of the preoperative knee. Furthermore, the change in rotational knee kinematics after BCR-TKA showed associations with clinical outcomes.

Regarding intraoperative rotational kinematics, Hamada et al. reported the results of a cadaveric study using the same navigation system as ours [15]. They showed that the rotational kinematic pattern of a BCR-TKA-treated knee was different from those of the native knee and menisectomy-treated knee: 1) the native knee showed a gradual internal rotation throughout flexion, 2) the menisectomy-treated knee showed a gradual increase in tibial internal rotation during flexion between 0° and 20° and > 80° and a gradual decrease between 20° and 80° of flexion, and 3) the BCR-TKA-treated knee showed a gradual decrease in the tibial internal rotation during flexion between 0° and 90° and a gradual increase of > 90° [15]. The rotational kinematic pattern of the menisectomy-treated knee demonstrated in the previous report was very close to the kinematic pattern of our preoperative knees. One of the possible reasons was that we registered the anatomical landmarks using the pointer and measured intraoperative kinematics after cutting the anterior horn of the medial and lateral menisci. In addition, most of the BCR-TKA-treated knees had severe meniscal damage; however, the chondral damage and joint deformity were not extremely severe. Conversely, the rotational kinematic pattern of the BCR-treated knee shown in the previous report was different from that of our BCR-treated knees. One of the probable reasons was that in the present study, the surgery was performed using anatomically shaped prosthesis with the FA method, whereas the surgery in the previous study used nonanatomically shaped prosthesis (Vanguard XP, Zimmer Biomet, Warsaw, IN, USA) with mechanical alignment. The Journey II XR prosthesis used in the present study had an anatomical design featuring a femoral cruciate-retaining implant and a non-symmetrical tibial baseplate with an asymmetric notch that was positioned more anteriorly on its medial side to accept the ACL footprint, and it provided greater coverage while not limiting the capacity for rotation. The geometry of the articular surface had a greater effect on the tibiofemoral rotational kinematics [31]. Several studies have demonstrated that the knee kinematics after BCR-TKAs using nonanatomically designed prosthesis were not the same as normal knees and that ACL forces were higher than that of normal knees [32–34]. FA BCR-TKA using an anatomically designed prosthesis might lead to normal-like rotational kinematic patterns shown in this study.

However, the “screw home movement” that was reported as a sharp internal rotation near extension [35] was not reproduced well after BCR-TKA (Fig. 3). The screw-home mechanism was attributed to the function of the ACL and asymmetry between the medial and lateral femoral condyles. This movement is reported to be absent after most TKAs, except for bicruciate stabilized (BCS)-TKA [36–38]. Wada et al. showed that rotational kinematics including screw home movement was preserved after lateral UKA but not after medial UKA [39]. They suggested that changes in the articular surface geometry of the tibia in the medial compartment of the knee have a more significant effect on the screw-home mechanism than changes in the lateral compartment. The surface geometry of the polyethylene insert of the medial compartment was reported to be crucial in the restoration of normal rotational kinematics after BCR-TKA [31]. The polyethylene of Journey II XR was designed to mimic the anatomical geometry. Because the meniscus is absent after BCR-TKA, an updated design including the role of the meniscus might be necessary for more normal-like knee kinematics.

Regarding the relationship between the change in rotational kinematics after BCR-TKA and clinical outcomes, the more the internal rotation of the tibia relative to the femur at maximum flexion angle decreases, the lower the KOOS scores of the ADL subscale and improvement of KOOS symptom and ADL subscales (Figs. 3 and 5). Several studies have assessed the extent of tibial internal rotation during flexion, and tibial internal rotation at the maximum flexion angle was reported to have some relationship with the postoperative clinical outcomes after posterior cruciate-retaining, posterior cruciate-stabilized, and BCS TKAs [38–40]. Kono et al. investigated the relationship between postoperative kinematics and outcomes in BCR-TKA using cluster analysis [41]. They found that patients with inferior clinical outcomes exhibited consistent external rotation of the femur throughout the range of motion compared to those with superior outcomes. Although not statistically significant, the tibial component also tended to be more internally rotated in the poor outcome group. These findings suggest that even subtle deviations in rotational alignment of either component may result in altered kinematics and poorer clinical outcomes. Considering the results of this study, tibial rotation at the maximum flexion angle is also important for BCR-TKA, and preserving the preoperative tibial rotational angle is necessary for excellent clinical outcomes. Conversely, this study also showed that the more the internal rotation angle increased at extension, the better the improvement of clinical outcomes. That might mean that the loss of screw-home movement increased the clinical outcomes after BCR TKA. The screw-home movement is facilitated by the ACL, the articular surface and meniscus of the normal knees. Screw-home like movement at early flexion angles is reported to improve of the KOOS pain subscale after BCS TKA [42]. In BCR TKA, the ligament force to the ACL is reported to be highest at extension and the postoperative ligament force is larger than preoperative force [43]. Therefore, an increase of the internal rotation angle at extension might decrease the excessive ligament force to the ACL and improve the clinical outcomes. More studies are necessary to determine the most suitable rotational kinematics of BCR-TKA. Therefore, judging from the findings of the current study, we think surgeons should measure the rotational kinematics using the navigation or the robot system and pay attention to the rotational kinematic change after placing the trial implant. And if the rotational angle of at extension is comparable to that observed preoperatively, external rotation of the tibial component or internal rotation of the femur or a tight extension gap might be suspected. Before placing the final implant, it is necessary to adjust the rotational alignment or fine-tune the soft tissue balance as needed.

The AP translation of the femoral center after BCR-TKA with respect to the tibial center was similar to that of the preoperative knee, although the AP position of the femur after BCR-TKA was a little more posterior than that of the preoperative knee at 60° and 90° of flexion. No significant difference in anterior translation was found at full extension and 30° of flexion between the preoperative knee and the BCR-TKA-treated knee. As the normal ACL is most active from full extension to 30° flexion and resists anterior translation of the tibia [44, 45], ACL functions were well preserved after BCR-TKA. In addition, the AP position at maximum flexion angle did not change after BCR-TKA either. Although femoral rollback is observed at high flexion angles, normal ACL and PCL functions are needed to flex the knee while maintaining normal femoral and tibial bone positioning [46]. Therefore, the functions of both ACL and PCL were thought to be well preserved after FA BCR-TKA.

The AP position of the femur after BCR-TKA was a little more posterior than that of the preoperative knee at 60° and 90° flexion, although it did not cause poor clinical outcomes. Most studies about the AP position of the femur after TKA have shown the difference from the native and preoperative knees [47–49]. The AP position of BCR-TKA was reported to be similar to the native knee because of the characteristic structure, including an anterior post-cam design and asymmetric tibial surface; however, the femoral center was reported to be anteriorly located in the middle flexion ranges [49]. The anterior position of the femur might cause the maltracking of the patella and the increased pressure on the patellofemoral (PF) joint. Conversely, the femoral center of the BCR-TKA in the middle flexion ranges of the present study was a little posteriorly located, which will not lead to PF problems, and surgeons might not need to replace the patella when performing BCR-TKA.

This study has several limitations. First, this was a retrospective study. Second, the follow-up period was relatively short; thus, further long-term investigations are desired. Third, a relatively small number of patients were evaluated. Fourth, this study included only varus-type OA knees, and valgus-type cases were excluded due to limited numbers (only seven cases). Therefore, the generalizability of our findings to valgus-aligned knees is limited, and further studies including more valgus cases are warranted. Fifth, preoperative knees were different from normal healthy knees, although the deformity was not extremely severe. Sixth, four knee surgeons performed BCR TKA procedures. Although a senior surgeon participated in all procedures, the reproducibility of the surgical technique is unknown. Seventh, preoperative knee kinematics in the current study are not the kinematics of normal knees but the kinematics of OA knees. Additionally, all the kinematics measurements in the current study were performed under anesthesia using passive movement and not the active motion postoperatively, which differs from active, weight-bearing motion postoperatively. Thus, the clinical implications of the observed kinematic changes should be interpreted with caution, and further research is needed to clarify their relationship with in vivo functional performance. Eighth, although there have been many reports that showed the kinematic analysis using the same navigation system, it is difficult to know the accuracy of the kinematic analysis because the details of the true algorithm of measurements are not disclosed by the manufacturer [10, 15, 29, 31, 38–40]. Finally, the thresholds for KOOS subscales after TKA were reported as follows: pain, 8–16; symptoms, 6–15; ADL, 10–16; sport/recreation, 9–16; and quality of life, 8–14 [50–52]. And the regression line is y = − 0.9x + 91.2 in Fig. 5. Therefore, the value of the y-intercept varies from 80.9 to 93.9 because the x varies from − 3 to 11.5. Although this difference might be comparable to the MCID of ADL after TKA, it is certainly small, and this is one of the limitations of the current study.

## Conclusions

FA BCR using anatomically designed prosthesis showed good rotational kinematic patterns, although screw home movement was not reproduced well. The AP translation of the femur after BCR-TKA with respect to the tibia was similar to that of the preoperative knee. The change in rotational knee kinematics after BCR-TKA was associated with the clinical outcomes. A positive relationship was found between the difference in rotational angles at 0° and improvement of KOOS scores, and a negative relationship was observed between the difference in rotational angles at maximum flexion angle and KOOS scale improvement.

## Data Availability

The datasets used during the current study are available from the corresponding author on reasonable request.
